# Supplementary material for: Development and Validation of a Predictive Tool for Postpartum Hemorrhage after Vaginal Delivery: A Prospective Cohort Study
Source: Biology (Basel). 2022 Dec 29;12(1):54. doi: 10.3390/biology12010054 (PMC9855728; doi:10.3390/biology12010054)
Supplement: Supplementary file 1 [file biology-12-00054-s001.zip › biology-2074967-supplementary.pdf]

Supplementary materials:

Table S1: Inclusion frequency of bootstrap selection variables

Variables	Inclusion frequency (%)	Variables	Inclusion frequency (%)
		Uterine myoma	32,73
**APTT ratio**	**92,23**	Previous PPH	35,46
**Antepartum bleeding**	**91,26**	Smoking during pregnancy	24,43
**Pre-eclampsia**	**89,29**	White blood cells	45,26
**Platelet**	**92,63**	Neutrophils	24,90
**Labor duration**	**87,08**	Arterial disease	34,78
**Multiple pregnancy**	**81,76**	Haemoglobin	37,08
**Macrosomia ***	**81,41**	Alcohol during pregnancy	33,89
Assisted reproductive technology	78,19	Autoimmune disease	19,78
Ethnicity	55,25	Diabetes mellitus	29,55
Fibrinogen	76,63	Temperature	40,62
Infectious disease	65,39	Transfusion history	23,69
Term of delivery	65,41	Monocytes	25,00
Anti-inflammatory drugs	68,75	Prothrombin time	24,34
Age	66,82	Blood group	20,86
Immature platelet fraction	61,96	Cardiac disease	24,24
Placenta insertion	60,15	Hydramnios	21,82
Previous C-section	59,39	Intrahepatic cholestasis	23,04
Body mass index	70,51	Parity	20,50
Fibrin monomers	56,92	Obstetrical bleeding history	19,04
Premature rupture of membranes	52,63	Lymphocytes	23,79
Gestational hypertension	44,45	Antiplatelets	18,85
Anticoagulants	54,24	Psychiatric drugs	23,90
D-Dimers	59,60	Gestational diabetes	19,85
Mean corpuscular volume	39,98	Intrauterine growth restriction	23,19
Anesthesia	38,41	Venous thromboembolism history	26,12
Instrumental birth	43,43	Hematocrit	28,65
Labor induction	43,39	Gynecological bleeding history	19,75
Weight gain	39,33	Premature delivery threat	16,21
Basophils (G/l)	24,61	Eosinophils	2,43
Nephrological disease	40.15		

** Macrosomia was defined as a birth weight >4000 grams*

PPH: postpartum hemorrhage; C-section: Cesarean section; APTT: Activated Partial Thromboplastin Time

Figure S1: ROC curves of predictive score for PPH (bootstrap simulated samples)

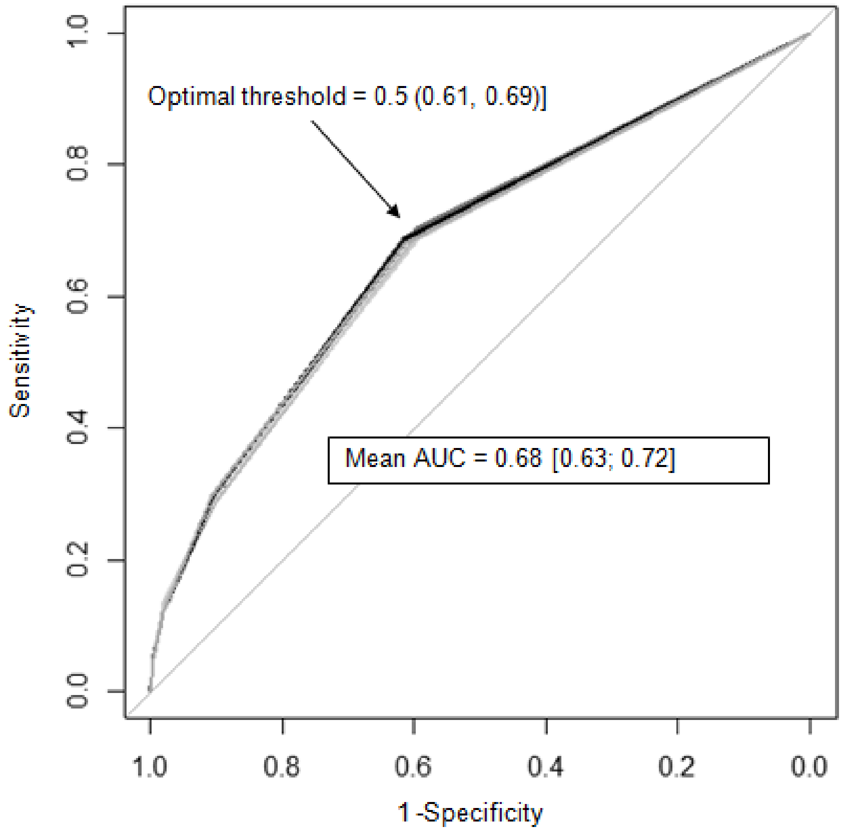

Figure S2: ROC curve of predictive score for PPH on the validation cohort

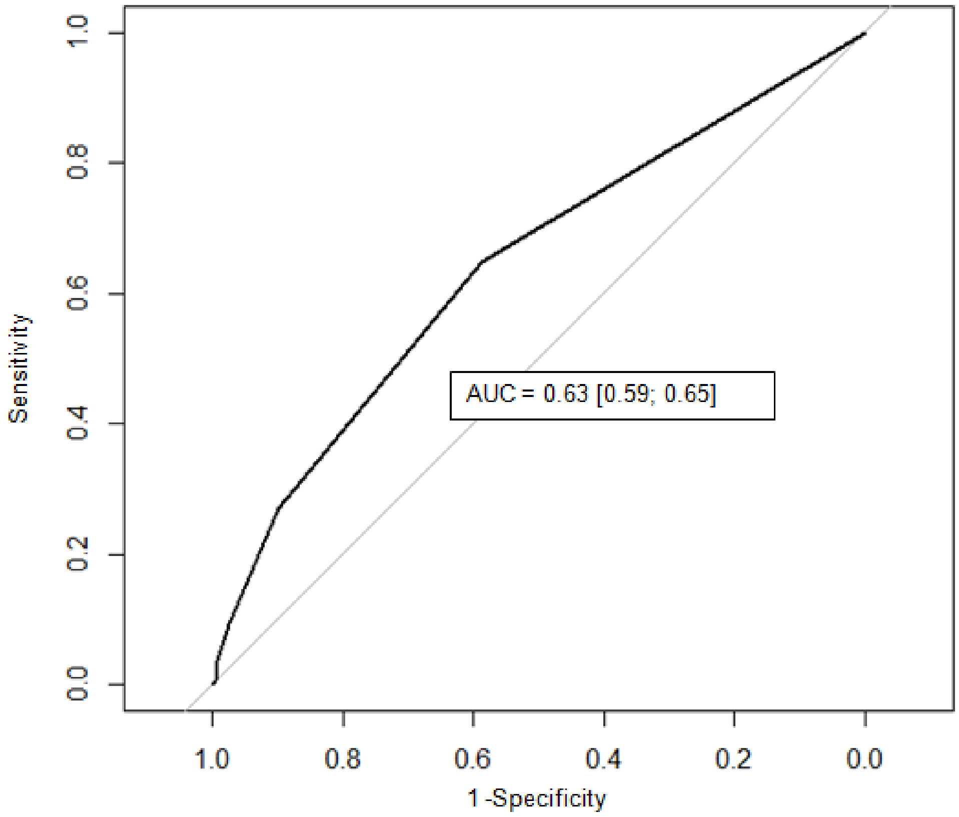

Table S2: Automatic spreadsheet to calculate the exact probability of PPH risk

Probability of postpartum hemmorhage risk

	Yes/No ?
**CLINICAL PARAMETERS**	
Pre-eclampsia	Yes
Antepartum bleeding	No
Multiple pregnancy	Yes
Labor duration ≥ 8 hours	No
Macrosomia *	Yes
**BIOLOGICAL PARAMETERS**	
Platelets < 150 Giga/l	No
APTT ratio ≥ 1.1	No
	**PPH probability : 52.8 %**

** Macrosomia was defined as a birth weight >4000 grams*
PPH: postpartum hemorrhage; aPTT: Activated Partial Thromboplastin Time
